# Deep Learning for the Diagnosis of Esophageal Cancer in Endoscopic Images: A Systematic Review and Meta-Analysis

**DOI:** 10.3390/cancers14235996

**Published:** 2022-12-05

**Authors:** Md. Mohaimenul Islam, Tahmina Nasrin Poly, Bruno Andreas Walther, Chih-Yang Yeh, Shabbir Seyed-Abdul, Yu-Chuan (Jack) Li, Ming-Chin Lin

**Affiliations:** 1 Graduate Institute of Biomedical Informatics, College of Medical Science and Technology, Taipei Medical University, Taipei 110, Taiwan; 2 International Center for Health Information Technology (ICHIT), Taipei Medical University, Taipei 110, Taiwan; 3 Research Center of Big Data and Meta-Analysis, Wan Fang Hospital, Taipei Medical University, Taipei 116, Taiwan; 4 Deep Sea Ecology and Technology, Alfred-Wegener-Institut Helmholtz-Zentrum für Polar- und Meeresforschung, Am Handelshafen 12, D-27570 Bremerhaven, Germany; 5 Department of Dermatology, Wan Fang Hospital, Taipei 116, Taiwan; 6 TMU Research Center of Cancer Translational Medicine, Taipei Medical University, Taipei 110, Taiwan; 7 Department of Neurosurgery, Shuang Ho Hospital, Taipei Medical University, New Taipei City 23561, Taiwan; 8 Taipei Neuroscience Institute, Taipei Medical University, Taipei 11031, Taiwan

**Keywords:** artificial intelligence, convolutional neural network, gastrointestinal endoscopy, esophageal cancer, automated diagnosis

## Abstract

**Simple Summary:**

Esophageal cancer is the seventh leading cause of cancer-related mortality worldwide, with a 5-year survival rate of around 20%. Recently, deep learning (DL) models have shown great performance in image-based esophageal cancer diagnosis and prognosis prediction. In this study, a comprehensive literature search was conducted on studies published between 1 January 2012 and 1 August 2022 from the most popular databases, namely, PubMed, Embase, Scopus, and Web of Science. This study, thus, systematically summarizes the application of a DL model for esophageal cancer diagnosis and discusses the potential limitations and future directions of DL techniques in esophageal cancer therapy.

**Abstract:**

Esophageal cancer, one of the most common cancers with a poor prognosis, is the sixth leading cause of cancer-related mortality worldwide. Early and accurate diagnosis of esophageal cancer, thus, plays a vital role in choosing the appropriate treatment plan for patients and increasing their survival rate. However, an accurate diagnosis of esophageal cancer requires substantial expertise and experience. Nowadays, the deep learning (DL) model for the diagnosis of esophageal cancer has shown promising performance. Therefore, we conducted an updated meta-analysis to determine the diagnostic accuracy of the DL model for the diagnosis of esophageal cancer. A search of PubMed, EMBASE, Scopus, and Web of Science, between 1 January 2012 and 1 August 2022, was conducted to identify potential studies evaluating the diagnostic performance of the DL model for esophageal cancer using endoscopic images. The study was performed in accordance with PRISMA guidelines. Two reviewers independently assessed potential studies for inclusion and extracted data from retrieved studies. Methodological quality was assessed by using the QUADAS-2 guidelines. The pooled accuracy, sensitivity, specificity, positive and negative predictive value, and the area under the receiver operating curve (AUROC) were calculated using a random effect model. A total of 28 potential studies involving a total of 703,006 images were included. The pooled accuracy, sensitivity, specificity, and positive and negative predictive value of DL for the diagnosis of esophageal cancer were 92.90%, 93.80%, 91.73%, 93.62%, and 91.97%, respectively. The pooled AUROC of DL for the diagnosis of esophageal cancer was 0.96. Furthermore, there was no publication bias among the studies. The findings of our study show that the DL model has great potential to accurately and quickly diagnose esophageal cancer. However, most studies developed their model using endoscopic data from the Asian population. Therefore, we recommend further validation through studies of other populations as well.

## 1. Introduction

Rationale: Esophageal cancer is one of the most commonly diagnosed adenocarcinomas globally, with an estimated 0.5 million new cases annually [1]. The prognosis of esophageal cancer is poor and is the sixth leading cause of cancer-related mortality worldwide, with over 0.5 million deaths annually [2]. The 5-year overall survival rate of early EC is only 20% [3]; however, the survival rate depends on several factors, including the stages of esophageal cancer. The 5-year survival rate of localized (confined to the primary site) esophageal cancer is 46.4%, whereas the relative survival of distant esophageal cancer (spread to lymph nodes) is 5% [4]. The two primary histologic subtypes of esophageal cancer are esophageal squamous cell carcinoma (ESCC) and esophageal adenocarcinoma (EAC), which contribute approximately 90 percent of total esophageal cancer [5]. Although the prevalence of ESCC is always high, recent years have witnessed an increasing trend of EAC in the United States of America (USA) and other Western countries [6,7]. Previous studies reported that patients with EAC have a better overall median survival than ESCC, particularly in early stage disease [8,9]. The risk factors of ESCC include smoking, alcohol, dietary, and male gender, whereas gastro-esophageal reflux disease (GERD) and obesity are two major risk factors for EAC [10,11]. Barrett’s esophagus (BE) is also an established premalignant lesion [12,13], which increases the risk of EAC up to 40-fold [14].

An early and accurate diagnosis of esophageal cancer is essential in determining the appropriate management of esophageal cancer patients and improving their overall survival rate [15]. Esophageal cancer is often detected in the advanced stage, which requires highly invasive treatments such as surgical resection and chemoradiotherapy [16,17]. The early detection of esophageal cancer through widely used screening programs has shown its effectiveness in reducing esophageal cancer-related mortality and improving the overall survival rate. The introduction of image-enhanced endoscopies, such as narrow-band imaging (NBI) and white-light imaging (WLI), has improved the early detection rate of esophageal cancer [18,19,20]. However, esophageal cancer detection is always challenging and depends on substantial expertise and experience [21]. Recently, DL models, especially the convolutional neural network (CNN) model, have performed remarkably well in various medical fields, including esophageal cancer diagnosis and prognosis [22].

Goal: Notwithstanding the growing interest in and opportunities for the application of the DL model for the diagnosis of esophageal cancer using endoscopic images, previous studies have not comprehensively reviewed the extant literature reporting on the application of the DL model to diagnose esophageal cancer. If the DL algorithm might be considered in the future for use in a real-world clinical setting, the performance of this algorithm should first undergo the same degree of assessment as current practices to evaluate their acceptability. Therefore, in the present study, we aimed to conduct a systematic review and meta-analysis from studies that applied the DL to determine the diagnostic performance for esophageal cancer.

## 2. Methods

### 2.1. Research Design

This systematic review was conducted according to the Preferred Reporting Items for Systematic Reviews and Meta-Analysis of Diagnostic Test Accuracy Studies (PRISMA-DTA) [23]. The study has not been registered.

### 2.2. Search Methods for Identification of Studies

#### Electronic Database Search

A search of PubMed, EMBASE, Scopus, and Web of Science, between 1 January 2012 and 1 August 2022, was conducted to identify potential studies evaluating the diagnostic performance of the DL model for the diagnosis of esophageal cancer using endoscopic images, with the assistance of experts in systematic reviews and meta-analysis. We used appropriate MeSH (Medical Subject Headings) terms as given below: “Deep learning” OR “computer-aided system” OR “convolutional neural network/s” AND “esophageal cancer” OR “esophageal neoplasm” OR “esophageal adenocarcinoma” OR “Barrett’s esophagus”.

### 2.3. Inclusion and Exclusion Criteria

Studies were included if they satisfied all the following criteria: (a) studies evaluated the diagnostic test accuracy of DL for esophageal cancer using endoscopic images, (b) studies provided sensitivity, specificity, and accuracy, or studies provided adequate information to calculate these data, (c) a prospective or retrospective study design, (d) provided appropriate information regarding inclusion and exclusion criteria, and (e) studies were published in English. However, studies were excluded if any of the following criteria were met: (a) studies published in the form of review, letter, or case report, (b) studies used the same database (we only included recent studies), and (c) studies did not provide any information regarding the number of patients or images. Two authors (M.M.I and T.N.P.) independently assessed the eligibility criteria of all the retrieved studies.

### 2.4. Data Extraction

The same two authors read all the selected studies carefully and extracted the following information using a standardized form: (1) study characteristics (authors, country of origin, year of publication, total number of endoscopic images, and study design), (2) demographic characteristics (gender and number of patients), (3) model characteristics (algorithms, data partition, and model description), (4) results (sensitivity, specificity, area under receiver operating characteristic curve, positive predictive value, and negative predictive value).

### 2.5. Quality Assessment

The same two authors also evaluated the methodological quality of the selected studies using established questionnaires and criteria established in the Quality Assessment of Diagnostic Accuracy Studies-2 (QUADAS-2) [24]. This tool is widely accepted for assessing the risk of bias and the applicability of diagnostic studies. QUADAS-2 comprises four main domains: (a) patient selection, (b) index test, (c) reference standard, and (d) flow and timing.

### 2.6. Statistical Analysis

The diagnostic performance of DL for detecting esophageal cancer was the primary outcome of our meta-analysis. We calculated the pooled sensitivity and specificity with 95% confidence intervals (CIs) using the bivariate random effects model [25,26,27]. However, the random effects model by DerSimonian and Laird was used to calculate the independent proportions and their differences [28]. A summary receiver operating characteristic (SROC) curve with a 95% confidence region was plotted to visualize the study findings. We also determined the heterogeneity of the study‘s findings using the inconsistency index (I^2^) as follows: 0% to 25%, might not be low; 25% to 50%, considered as low; 50% to 75%, medium heterogeneity; and 75% to 100%, considerable heterogeneity [29,30]. We also calculated the positive likelihood ratio, negative likelihood ratio, and diagnostic odd ratio (Supplementary Materials S1) [21]. *p* values < 0.05 were considered statistically significant. R (R Core Team and the R Foundation for Statistical Computing, version: 4.2.1) and MedCalc (MedCalc Software Ltd, Ostend, Belgium) were used to perform all statistical analyses.

## 3. Results

### 3.1. Study Selection

A total of 2491 studies were retrieved during the initial search, after which 1375 duplicate studies were excluded. From 1116 nonduplicate studies, 1088 were excluded after reviewing the titles and abstracts. However, 39 studies underwent full-text evaluation. Afterward, eleven studies were excluded due to review, not using the DL model, and insufficient data. The remaining 28 studies were selected for inclusion in the meta-analysis [22,31,32,33,34,35,36,37,38,39,40,41,42,43,44,45,46,47,48,49,50,51,52,53,54,55,56,57] (Supplementary Mayerials Figure S1).

### 3.2. Study Characteristics

All of the studies used in this systematic review and meta-analysis are presented in Table 1. The selected studies were published between 2019 [57] and 2022 [31] with a total of 703,006 endoscopic images, and each study followed the standard protocol to detect esophagus cancer. The included images were captured by white-light imaging (WLI), narrow-band image (NBI), or blue light image (BLI). Expert endoscopists checked the appropriateness of images and marked images as cancerous and normal. Most of the studies were retrospective study designs, except for three studies. However, all studies used the CNN model to train and validate their findings. Twenty-three studies were conducted on Asian populations, while only five studies were conducted on Western populations. Esophageal squamous cell cancer (ESCC) was the primary target for eighteen studies, while six studies reported Barret’s esophagus (BE) and four studies showed esophageal adenocarcinoma (EAC), including ESCC as the outcome. Nine studies developed a DL model using WLI, while six studies included NBI, and eleven studies utilized both WLI and NBI. Moreover, one study trained a DL model for the diagnosis of esophageal cancer using BLI, while another study trained their DLF model using volumetric laser endomicroscopy (VLE) [53] and endocytoscopic system image (ECS) [52].

### 3.3. Deep Learning Model for Esophageal Cancer Diagnosis

**Figure 1:**
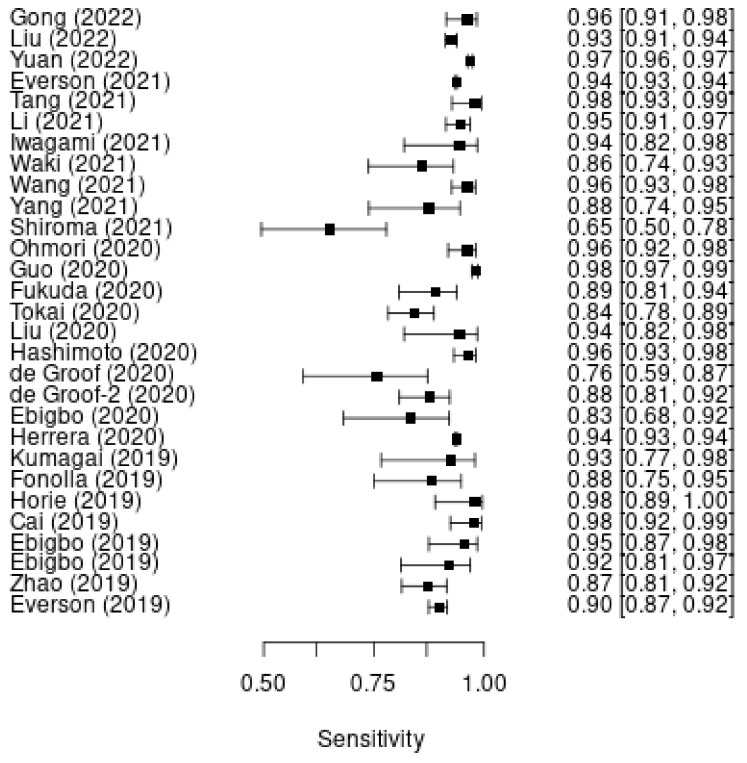
Pooled sensitivity of the DL model for the diagnosis of esophageal cancer.

**Figure 2:**
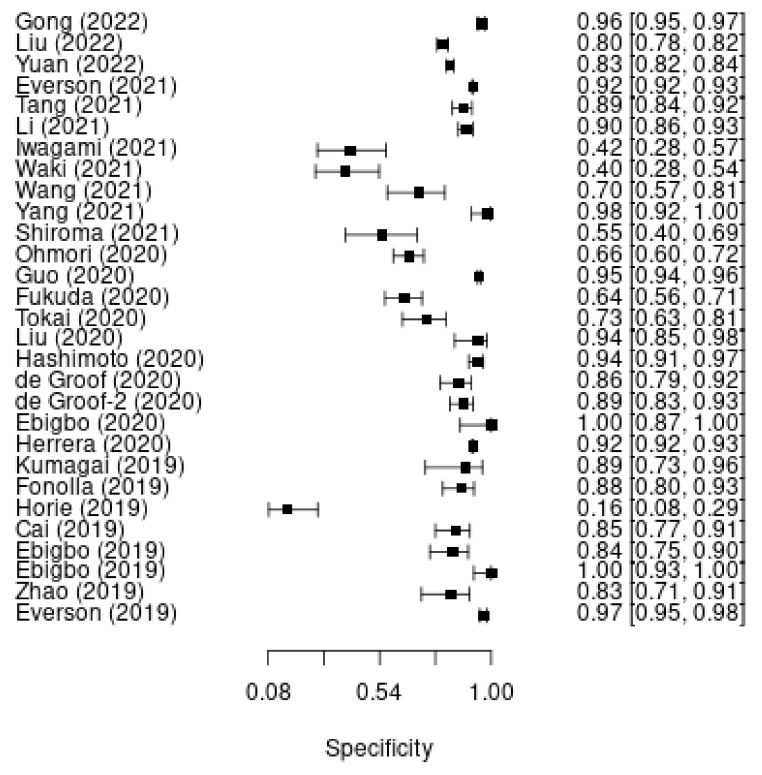
Pooled specificity of the DL model for the diagnosis of esophageal cancer.

A total of 28 studies were included in our study. The pooled sensitivity for the diagnosis of esophageal cancer was 93.80% (95% CI: 93.64–93.96%; Figure 1). The pooled specificity for the diagnosis of esophageal cancer was 91.73% (95% CI: 91.52%–91.94%; Figure 2). There was significant heterogeneity among the studies (I^2^ = 86.6%, χ2 = 268.56, *p* < 0.001, and I^2^ = 97.6%, χ2 = 1176.58, *p* < 0.001).

**Figure 3:**
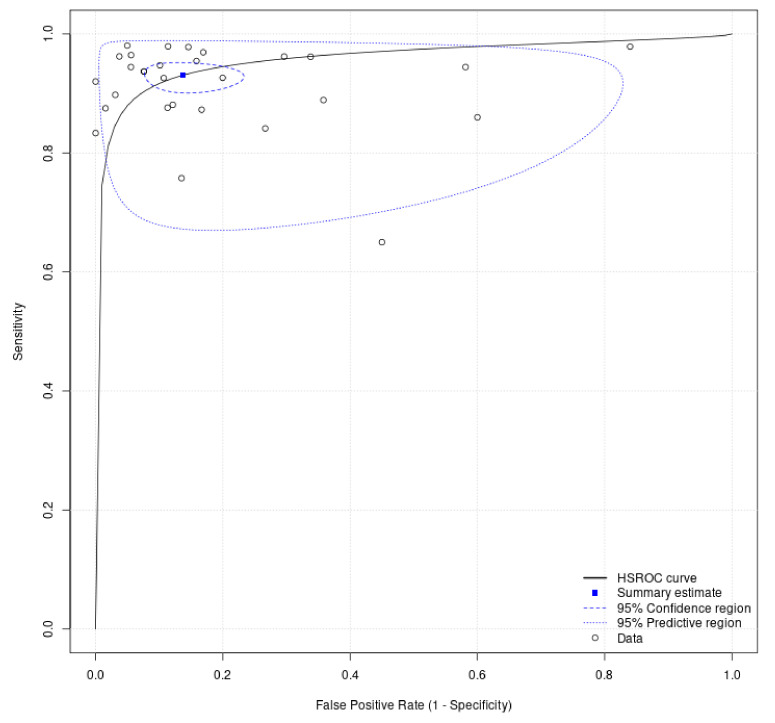
The summary of the area under the receiver operating curve for diagnosis of esophageal cancer.

However, the pooled positive likelihood ratio, negative likelihood ratio, and diagnostic odds ratio for the diagnosis of esophageal cancer were 6.41 (95% CI: 4.74–8.67), 0.09 (95% CI: 0.08–0.11), and 81.89 (95% CI: 61.90–108.34), respectively. There was significant heterogeneity among the studies (I^2^ = 99.0%, τ^2^ = 59.05, Cochrane-Q = 2865.73, *p* < 0.001; I^2^ = 91.4%, τ^2^ = 0.08, Cochrane-Q = 327.37, *p* < 0.001; I^2^= 94.7%, τ^2^ = 0.34, Cochrane-Q = 528.97, *p* < 0.001). The AUROC of the DL was 0.96 (95%CI: 0.94–0.98; Figure 3).

### 3.4. Subgroup Analysis

We also conducted subgroup analyses based on the region, study design, endoscopy types, histological types, and methodological quality (Table 2). Nine studies evaluated the diagnostic performance of the DL model for the diagnosis of esophageal cancer using WLI. The pooled sensitivity and specificity of the DL model for esophageal cancer were 92.60% (95% CI: 91.39%–93.69%) and 86.95% (95% CI: 85.58%–88.25%). Six studies used NBI to train and validate the DL model for esophageal cancer diagnosis. The pooled sensitivity and specificity of the DL model for esophageal cancer were 93.73% (93.56%–93.89%) and 92.66% (95% CI: 92.45%–92.86%).

While comparing the diagnostic performance between high- and low-quality studies, the diagnostic performance of the DL model for high-quality studies was higher than that for low-quality studies. The pooled accuracy, sensitivity, and specificity of high-quality studies were 92.96% (95% CI: 92.83%–93.09%), 93.85% (95% CI: 93.69%–94.01%), and 91.80% (95% CI: 91.59%–92.01%), respectively. The pooled accuracy, sensitivity, and specificity of low-quality studies were 89.16% (95% CI: 87.88%–90.35%), 90.40% (95% CI: 88.70%–91.93%), and 87.75% (95% CI: 85.75%–89.57%), respectively.

### 3.5. External Validation

Five studies externally validated their model and evaluated the performance for the diagnosis of esophageal cancer. At external validation, the pooled accuracy of the DL model was 90.31% (89.42%–91.14%), while the corresponding sensitivity, specificity, positive predictive value, and negative predictive value for the diagnosis of esophageal cancer were 95.26% (95% CI: 94.37%–96.04%), 84.02% (95% CI: 82.37%–85.58%), 88.33% (95% CI: 87.27%–89.32%), and 93.31% (95% CI: 92.15%–94.31%).

### 3.6. Performance Comparison between DL and Endoscopists

**Figure 4:**
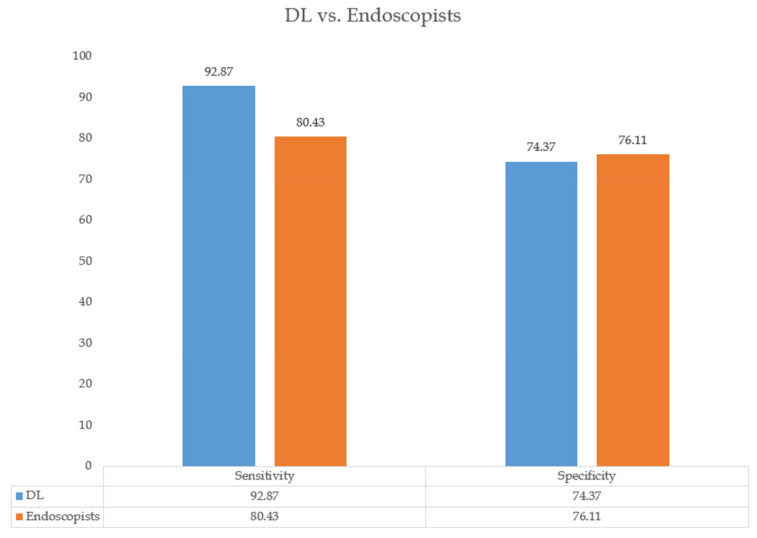
Diagnostic performance for esophageal cancer by the DL system vs. endoscopists.

Ten studies also compared the diagnostic performance of the DL model with that of endoscopists. The performance measures are presented in Figure 4. The sensitivity of the DL model for the diagnosis of early esophageal cancer exceeded those of endoscopists (92.87% vs. 80.43%). A further analysis of specificity showed that the DL model had a similar performance when compared with that of the endoscopists (74.37% vs. 76.11%).

### 3.7. Publication Bias

**Figure 5:**
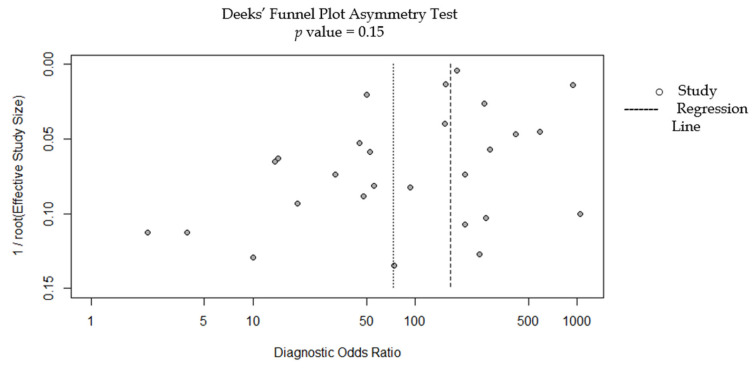
Deeks’ funnel plot of the performance of the DL model for the diagnosis of esophageal cancer.

Deeks’ funnel plot of the asymmetry test showed no evidence of publication bias (*p* = 0.15) (Figure 5).

## 4. Discussion

We conducted a meta-analysis to evaluate the performance of the DL model for the diagnosis of esophageal cancer using data from 28 studies. The overall pooled estimation showed that the DL performance for the diagnosis of esophageal cancer performed better in terms of sensitivity, specificity, and AUROC. To determine the generalizability of the DL model to external settings, the DL model also showed a better performance when used with different datasets. The findings of our study suggest that the accurate diagnosis performance of DL may help a physician to increase the early diagnosis of esophageal cancer and decrease mortality. Because the identification of early esophageal cancer has been a subject of concern and solely depends on expert endoscopists, the higher sensitivity and specificity of DL could correctly and accurately detect esophageal cancer in patients, provide supportive treatment, and improve patient outcomes.

Four previously published studies also assessed the impact of AI-assisted models for detecting esophageal cancer using endoscopic images [58,59,60,61]. Zhang et al. [58] included sixteen studies to provide scientific evidence for using AI-assisted models to detect esophageal neoplasm. The pooled sensitivity and specificity and AUROC of AI-assisted models for esophageal cancer detection were 0.94 (95% CI: 0.92–0.96), 0.85 (95% CI: 0.73–0.92), and 0.97 (95% CI: 0.95–0.98), respectively. They also reported that the performance of AI-based models was better than endoscopists in terms of the pooled sensitivity 0.94 [95% CI: 0.84–0.98] vs 0.82 [95% CI: 0.77–0.86]. Lui et al. [59] conducted a systematic review and meta-analysis to evaluate the diagnostic accuracy of AI models for gastric, esophageal neoplastic lesions, and *Helicobacter pylori* status. A total of 23 studies were included in that study; however, only 10 studies were used to evaluate early esophageal cancer detection. The pooled sensitivity, specificity, and AUROC on the detection of squamous esophagus neoplasm were 0.75 (95% CI: 0.48–0.92), 0.92 (95% CI: 0.66–0.99), and 0.88 (95% CI: 0.82–0.96), respectively. Bang et al. [60] included 21 studies in the systematic review. Among them, 19 studies were included in the meta-analysis to evaluate the diagnostic test accuracy of the deep learning or machine learning model of esophageal cancers. The pooled sensitivity, specificity, and AUROC of DL algorithms for the diagnosis of esophageal cancer were 0.94 (95% CI: 0.89–0.96), 0.88 (95% CI: 0.76–0.94), and 0.97 (95% CI: 0.95–0.99), respectively. Mohan et al. [61] performed a meta-analysis to examine the pooled performance rates for CNN-based AI in diagnosing gastrointestinal neoplasia from endoscopic images. Nineteen studies met all inclusion criteria for detecting gastrointestinal neoplasm; however, only five studies were used to evaluate the impact of CNN in diagnosing esophageal cancer. The pooled sensitivity, specificity, and accuracy of the CNN model for diagnosis of esophageal cancer were 0.87 (95% CI: 0.69–0.95), 0.87 (95% CI: 0.74–0.94), and 0.87 (95% CI: 0.76–0.93), respectively. This study included a higher number of studies to summarize the available evidence for the accuracy of the DL algorithm in diagnosing esophageal cancer. Moreover, this study showed not only sensitivity and specificity but accuracy and positive and negative predictive value, which are essential metrics for making clinical decisions. The findings of our study showed that the DL model could play a crucial role in diagnosing esophageal cancer in the near future when this algorithm might be employed in a busy daily clinical practice.

The detection rate of esophageal cancer is relatively poor (more than 40% percent of patients with esophageal cancer are detected at a late stage), and the 5-year survival rate is approximately 20% [62]; therefore, the early diagnosis of esophageal cancer is important for both clinicians and patients. The early diagnosis of esophageal cancer could assist clinicians in decision-making, improve patients’ management, and reduce healthcare costs. Endoscopy is one of the reliable methods for early esophageal cancer screening because of its high diagnostic performance [63]. Diagnostic accuracy depends on several factors such as the quality of images, instruments, and endoscopists. Traditional statistical models could hardly perform analyzing endoscopic images for the diagnosis of esophageal cancer; however, the DL model has been applied and has shown great performance in esophageal cancer diagnosis. To the best of our knowledge, this is the first comprehensive systematic review and meta-analysis of the diagnostic performance of the DL-based AI model for the diagnosis of esophageal cancer. The findings of this study demonstrate conclusively that the diagnostic performance of the DL model for the diagnosis of esophageal cancer using endoscopic images was clinically highly satisfactory in terms of sensitivity, specificity, and AUROC. For esophageal cancer diagnosis, DL achieved a pooled AUROC of 0.96 with a pooled sensitivity of 94% and a specificity of 92%. In terms of the generalizability of the DL model, the DL model also achieved good performance in the external validation.

The early detection of esophageal cancer offers a higher chance of survival [64]. Upper gastrointestinal endoscopy is still considered the standard method for the diagnosis of esophageal cancer [65,66]. Previous evidence shows that endoscopic techniques have made significant progress over the last decades [67,68,69]. WLI is widely used and the most basic endoscopy diagnostic modality to diagnose esophageal cancer [70]. However, the application of WLI for the diagnosis of esophageal cancer is limited because the DL model could not perform well. Therefore, the application of NBI and VLI has been increased for the early diagnosis of esophageal cancer. Consequently, researchers are now using both modalities together to develop DL models for identifying early esophageal cancer. The findings of our study show that the pooled sensitivity and specificity of the DL model, which used NBI images, had significantly better performance than those using WLI images.

There are several strengths and limitations associated with this meta-analysis. First, this is the first comprehensive study that summarized the performance of the DL model for the diagnosis of esophageal cancer using endoscopic images. Second, this study provided clinically important diagnosis metrics such as accuracy, positive predictive value, and negative predictive value, which may help a physician to make the appropriate decision for the patient with a high risk of esophageal cancer. Nevertheless, this study has some limitations that need to be addressed. First, most of the included studies were retrospective study designs. Although external validation and prospective evaluation showed great performance, more studies are needed that could evaluate the performance of the DL model using prospectively collected data or real-time evaluation. Second, two-thirds of the studies developed their model and tested the performance using the same continent’s population (namely, Asia); therefore, more data are warranted from other continents to validate the performance of the current model. Finally, heterogeneity among the studies was high, although it can be partially explained by regional effect, image quality, image modality, and histological types.

## 5. Conclusions

This is the first comprehensive systematic review and meta-analysis to show that the DL model was able to diagnose early esophageal cancer with high sensitivity and specificity. The performance of the DL model for esophageal cancer diagnosis was higher for NBI than for WLI. Most of the studies were from Asia and used a retrospective design; therefore, more prospective evaluations with various populations are warranted in the future.

## Figures and Tables

**Table 1:** The characteristics of the included studies.

Author	Year	Study Type	Country/ Region	Modality	Model	Total Images	Total Patient	Number of Endoscopists	Real-Time	Compare with Endoscopist	External Validation	Video Validation	Target	Quality
Gong	2022	RE	Korea	WLI	CNN	5162	NR	NR	No	No	Yes	No	EC	H
Liu	2022	RE	China	WLI	CNN	13,083	1239	14	No	Yes	Yes	No	ESCC	H
Yuan	2022	RE	China	WLI, NBI	CNN	53,933	2621	11	Yes	Yes	Yes	Yes	ESCC	H
Everson	2021	RE	Taiwan	NBI	CNN	67,742	NR	3	No	Yes	No	No	ESCC	H
Tang	2021	RE	China	WLI	CNN	4002	1078	10	Yes	Yes	Yes	Yes	ESCC	H
Li	2021	RE	China	WLI, NBI	CNN	4735	NR	20	No	Yes	No	No	ESCC	H
Iwagami	2021	RE	Japan	WLI, NBI	CNN	232	79	15	Yes	Yes	Yes	No	EAC	L
Waki	2021	RE	Japan	NBI/BLI	CNN	17,336	NR	21	Yes	Yes	No	Yes	ESCC	H
Wang	2021	RE	Taiwan	WLI, NBI	CNN	935	NR	NR	No	No	No	No	ESCC	L
Yang	2021	RE	China	WLI, NBI	CNN	13,297	6130	6	Yes	Yes	Yes	Yes	ESCC	H
Shiroma	2021	RE	Japan	NBI, WLI	CNN	8428	NR	18	Yes	Yes	No	Yes	ESCC	H
Ohmori	2020	RE	Japan	NBI, WLI, BLI	CNN	135	102	15	No	Yes	No	No	ESCC	H
Guo	2020	RE	China	NBI	CNN	6671	NR	NR	Yes	No	Yes	Yes	ESCC	H
Fukuda	2020	RE	Japan	NBI, BLI	CNN	238	NR	13	Yes	Yes	Yes	Yes	ESSC	L
Tokai	2020	RE	Japan	NBI, WLI	CNN	279	NR	13	No	Yes	No	No	ESCC	H
Liu	2020	RE	China	WLI	CNN	127	NR	NR	No	No	No	No	ESCC/EAC	H
Hashimoto	2020	RE	Japan	NBI, WLI	CNN	458	39	NR	Yes	No	No	No	BE	H
de Groof	2020	PR	Netherlands	WLI	CNN	144	20	NR	No	No	No	No	BE	H
de Groof	2020	RE	Netherlands	WLI	CNN	494,364	15,286	53	No	Yes	Yes	No	BE	L
Ebigbo	2020	RE	Europe	WLI	CNN	62	14	NR	No	No	No	No	BE	L
Herrera	2020	RE	Asia	NBI	CNN	67,742^$^	114	NR	NR	NR	No	No	ESCC	H
Kumagai	2019	RE	Japan	ECS	CNN	1520	55	NR	No	No	No	No	ESCC	H
Fonolla	2019	PR	Europe	VLI	CNN	141	NR	NR	No	No	No	No	BE	L
Horie	2019	RE	Japan	NBI, WLI	CNN	1118	97	NR	Yes	No	No	No	ESCC/EAC	H
Cai	2019	RE	China	WLI	CNN	187	52	16	No	Yes	No	No	ESCC	H
Ebigbo	2019	RE	Germany	NBI, WLI	CNN	148	NR	NR	No	No	No	No	BE/EAC	H
Ebigbo #	2019	RE	Germany	WLI	CNN	100	NR	NR	No	No	No	No	BE	H
Zhao	2019	RE	China	NBI	CNN	1383	NR	9	No	Yes	No	No	ESCC	H
Everson	2019	RE	Taiwan	NBI	CNN	7046	17	NR	No	No	No	No	ESCC	L

Note: RE—retrospective; PR—prospective; NBI—narrow-band imaging; WLI—white-light imaging; ECS—endocytoscopic system image; BLI—blue-laser image; VLI/E—volumetric laser image/endomicroscopy; NR—not reported; EC—esophageal cancer; EAC—esophageal adenocarcinoma; ESCC—esophageal squamous cell cancer; BE—Barret’s esophagus; CNN—convolutional neural network; H—high; L—low; and #—same study.

**Table 2:** Subgroup analyses for the diagnosis of esophageal cancer.

Subgroup	Studies (*n*)	Sensitivity (95%CI)	Specificity (95%CI)	Positive Predictive Value (95%CI)	Negative Predictive Value (95%CI)	Accuracy (95%CI)	Disease Prevalence
All	28	93.80 (93.64–93.96)	91.73 (91.52–91.94)	93.62 (93.47–93.77)	91.97 (91.77–92.15)	92.90 (92.77–93.03)	56.38 (56.14–56.63)
**Region**							
Asia	23	93.82 (93.66–93.98)	91.75 (91.55–91.96)	93.66 (93.51–93.80)	91.97 (91.77–92.16)	92.92 (92.80–93.05)	56.48 (56.23–56.72)
West	5	88.20 (84.39–91.36)	88.99 (86.03–91.51)	84.18 (80.66–87.16)	91.91 (89.51–93.79)	88.68 (86.41–90.68)	39.91 (36.68–43.21)
**Study design**							
Retrospective	25	93.82 (93.66–93.98)	91.75 (91.55–91.96)	93.65 (93.50–93.80)	91.97 (91.77–92.15)	92.92 (92.79–93.05)	56.46 (56.22–56.71)
Prospective	3	85.78 (80.23–90.27)	87.83 (84.10–90.95)	79.19 (74.26–83.38)	91.97 (89.08–94.14)	87.11 (84.12–89.73)	35.05 (31.17–39.08)
**Endoscopy type**							
WLI	9	92.60 (91.39–93.69)	86.95 (85.58–88.25)	85.42 (84.11–86.64)	93.44 (92.44–94.32)	89.51 (88.59–90.38)	45.22 (43.78 46.67)
NBI	5	93.73 (93.56–93.89)	92.66 (92.45–92.86)	94.39 (94.24–94.54)	91.81 (91.61–92.01)	93.27 (93.14–93.39)	56.85 (56.60–57.11)
Mixed (WLI + NBI)	11	95.70 (95.09–96.26)	80.99 (79.72–82.21)	86.15 (85.35–86.91)	93.85 (93.03–94.58)	89.12 (88.45–89.77)	55.27 (54.21–56.32)
VLE/BLI/ECS	3	88.24 (81.05–93.42)	74.58 (67.50–80.81)	70.00 (64.26–75.18)	90.41 (85.12–93.95)	80.07 (75.06–84.47)	40.20 (34.57–46.03)
**Histological type**							
ESCC	18	93.81 (93.65–93.97)	91.79 (91.58–92.00)	93.74 (93.59–93.89)	91.88 (91.69–92.07)	92.94 (92.81–93.06)	56.72 (56.47–56.96)
BE	6	90.87 (88.05–93.22)	91.41 (89.06–93.40)	88.80 (86.12–91.02)	93.04 (91.04–94.61)	91.18 (89.43–92.72)	42.85 (40.03–45.70)
EAC, including ESCC	4	95.80 (90.47–98.62)	52.38 (43.99–60.67)	61.96 (57.79–65.96)	93.90 (86.56–97.36)	71.80 (65.99–77.13)	44.74 (38.66–50.93)
**Methodological quality**							
High	21	93.85 (93.69–94.01)	91.80 (91.59–92.01)	93.68 (93.53–93.83	92.02 (91.82–92.21)	92.96 (92.83–93.09)	56.44 (56.19–56.69)
Low	7	90.40 (88.70–91.93)	87.75 (85.75–89.57)	89.27 (87.71–90.65)	89.03 (87.30–90.55)	89.16 (87.88–90.35)	52.98 (51.0–54.94)

## Supplementary Materials

The following supporting information can be downloaded at: https://www.mdpi.com/article/10.3390/cancers14235996/s1, Figure S1: PRISMA flowchart for identifying and selecting the study; Table S1: Evaluation metrics.
