# Supplementary material for: Deep Learning for the Diagnosis of Esophageal Cancer in Endoscopic Images: A Systematic Review and Meta-Analysis
Source: Cancers (Basel). 2022 Dec 5;14(23):5996. doi: 10.3390/cancers14235996 (PMC9736434; doi:10.3390/cancers14235996)
Supplement: Supplementary file 1 [file cancers-14-05996-s001.zip › cancers-1997715-supplementary.pdf]

Supplementary Materials: Artificial Intelligence in Upper Gastrointestinal Endoscopy: Application of a Convolutional Neural Network for Diagnosis of Esophageal Cancer in Endoscopic Images

Md. Mohaimenul Islam [1,2,3], Tahmina Nasrin Poly [1,2,3]; Bruno Andreas Walther [4], Chih-Yang Yeh [1], Syed Abdul Shabbir [1], Yu-Chuan (Jack) Li [1,2,3,5] and Ming Chin Lin

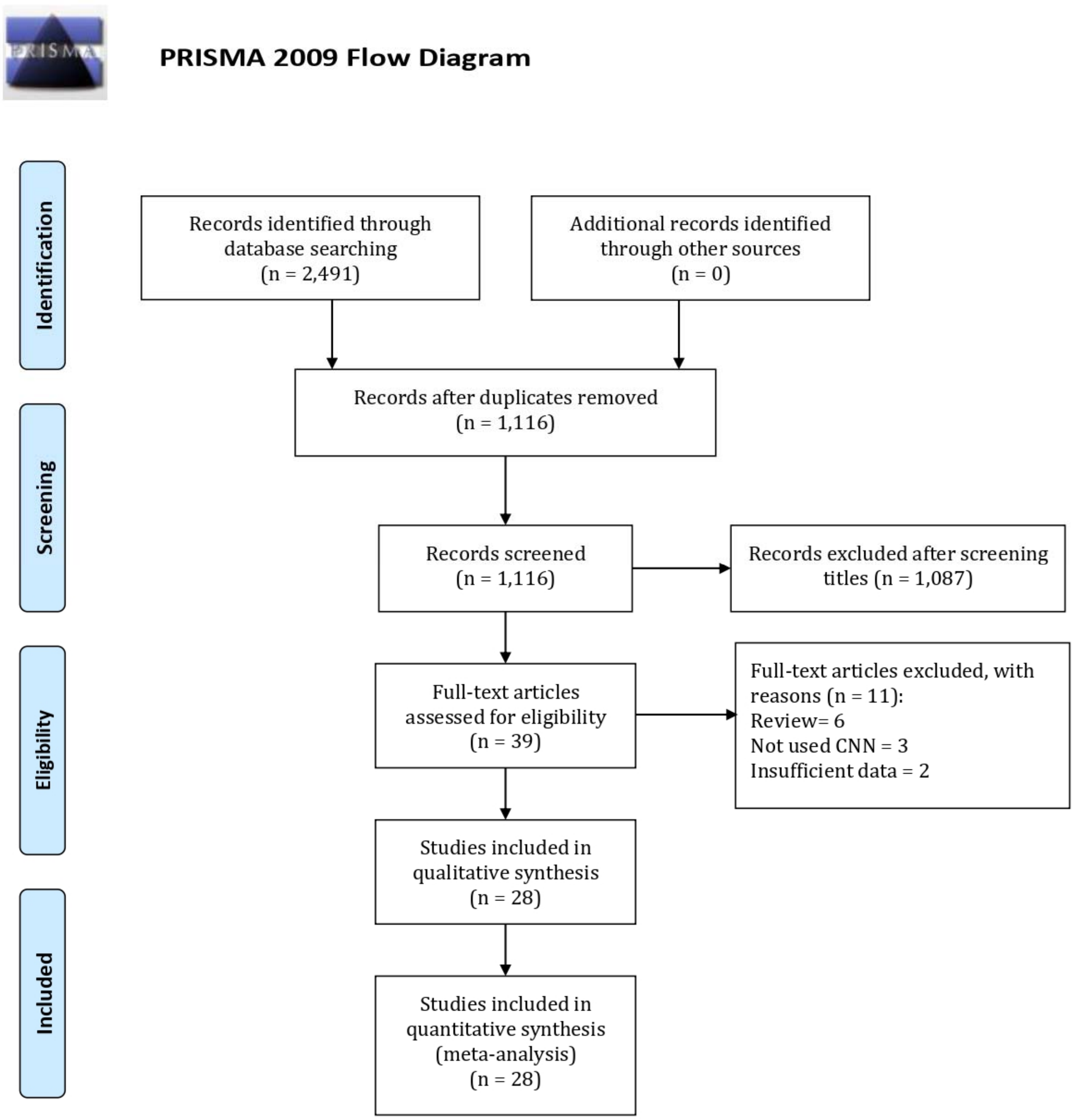

Figure S1. PRISMA flowchart for identifying and selecting study.

Supplementary Table S1. Evaluation metrics.

Evaluation metrics	Explanation	Formula
**Sensitivity**	It is the probability that a test will indicate 'esophageal cancer' among those with the disease	Sensitivity = True positive/ (True positive + False negative) × 100
**Specificity**	It is the fraction of those without the esophageal cancer who will have a negative test result	Specificity = True negative / (True negative + False positive) × 100
**Positive likelihood ratio**	ratio between the probability of a positive test result given the presence of the disease and the probability of a positive test result given the absence of the disease, i.e.	PLR = True positive rate / False positive rate = Sensitivity / (1-Specificity)
**Negative likelihood ratio**	ratio between the probability of a negative test result given the presence of the disease and the probability of a negative test result given the absence of the disease, i.e.	NLR = False negative rate / True negative rate = (1-Sensitivity) / Specificity
**Positive predictive value**	probability that the esophageal cancer is present when the test is positive	PPV = (Sensitivity × Prevalence) / [Sensitivity × Prevalence + (1-Specificity) × (1- Prevalence)
**Negative predictive value**	probability that the esophageal cancer is not present when the test is negative	NPV = [Sensitivity × (1 - Prevalence) / [(1- Sensitivity) × Prevalence + Specificity × (1- Prevalence)
**Accuracy**	overall probability that a patient is correctly classified	Accuracy = Sensitivity × Prevalence + Specificity × (1 − Prevalence)
